# Infection Associated with Global Cerebral Edema and Delayed Cerebral Ischemia in Patients with Aneurysmal Subarachnoid Hemorrhage

**DOI:** 10.3390/jcm14113808

**Published:** 2025-05-29

**Authors:** Daina Kashiwazaki, Kunitaka Maruyama, Saori Hamada, Shusuke Yamamoto, Emiko Hori, Naoki Akioka, Kyo Noguchi, Satoshi Kuroda

**Affiliations:** 1 Departments of Neurosurgery, Graduate School of Medicine and Pharmaceutical Sciences, University of Toyama, 2630 Sugitani, Toyama 930-0194, Japan; kmaru31@med.u-toyama.ac.jp (K.M.); saori115@med.u-toyama.ac.jp (S.H.); shuyama@med.u-toyama.ac.jp (S.Y.); emihori@med.u-toyama.ac.jp (E.H.); akioka@med.u-toyama.ac.jp (N.A.); skuroda@med.u-toyama.ac.jp (S.K.); 2 Departments of Radiology, Graduate School of Medicine and Pharmaceutical Sciences, University of Toyama, 2630 Sugitani, Toyama 930-0194, Japan; kyo@med.u-toyama.ac.jp

**Keywords:** cerebral edema, delayed cerebral ischemia, infection, subarachnoid hemorrhage

## Abstract

**Background/Objectives:** Patients with aneurysmal subarachnoid hemorrhage (SAH) experience functional impairment due to early brain injury and delayed complications. We aimed to clarify the association between cerebral edema and post-SAH infection. We investigated whether this association leads to delayed cerebral ischemia (DCI) and poor clinical outcomes. **Methods:** We included 189 patients diagnosed with aneurysmal SAH at our institution. Demographic data and data on World Federation of Neurological Surgeons (WFNS) grade, modified Fisher grade, aneurysm location, treatment methods, global cerebral edema (GCE) assessed according to Subarachnoid Hemorrhage Early Brain Edema Score (SEBES), DCI, infection, duration of hospital stay, and modified Rankin Scale at 3 months were collected. **Results:** Overall, 88 patients (46.6%) developed GCE ([SEBES] 3 or 4), while 101 patients (53.4%) did not. DCI was observed in 58 (30.7%) patients. Infectious complications occurred in 80 (42.3%) patients. Kaplan–Meier analysis results suggested a higher frequency of DCI among patients with GCE and infection than those without (*p* < 0.01). Logistic regression analysis identified GCE (*p* < 0.001, odds ratio [OR] 3.3, 95% confidence interval [CI] [1.3–8.6]), older age (*p* = 0.02, OR 2.5, 95%CI [1.2–4.9]), higher WFNS grade (*p* = 0.01, OR 3.9, 95%CI [1.5–9.5]), and mechanical ventilation use (*p* = 0.04, OR 1.4, 95%CI [1.1–3.9]) as risk factors for infection, while age (*p* = 0.03, OR 2.3, 95%CI [1.1–4.6]), WFNS grade (*p* < 0.001, OR 4.5, 95%CI [1.5–9.2]), and GCE + infection (*p* < 0.001, OR 4.1, 95%CI [1.3–8.9]) were independent risk factors for DCI. **Conclusions:** GCE–infection linkage is associated with DCI, poor clinical outcomes, and longer hospital stays in patients with aneurysmal SAH. Therefore, the EBI-DCI chain plays an important role in the postsurgical management of these patients.

## 1. Introduction

Subarachnoid hemorrhage (SAH) is a devastating type of stroke mainly caused by aneurysm rupture, and it is associated with a high morbidity and mortality [1]. Despite the development of early surgical clipping and endovascular treatment, patients with SAH experience functional impairments due to early brain injury (EBI) and delayed complications [2]. The mechanisms underlying secondary brain injury after SAH are multifactorial. EBI is a pivotal factor that leads to this outcome after SAH. EBI involves a variety of physiological disorders, including neuroinflammatory injury, blood–brain barrier disruption, and nerve cell apoptosis. Neuroinflammation plays a vital role in EBI [3,4,5]. Global cerebral edema (GCE) is a radiological surrogate marker of EBI. GCE is thought to be caused by rebound hyperemia associated with blood–brain barrier disruption in the setting of abnormal autoregulation after an initial increase in intracranial pressure, followed by intracranial circulatory arrest, which may trigger early GCE through cytotoxic edema [6]. Furthermore, due to post-SAH immunodepression, more than one-third of patients with SAH develop systemic or local infections [7]. Moreover, a high proportion of patients with SAH experience life-threatening complications such as vasospasm or delayed cerebral ischemia (DCI). Various biomarkers related to the pathophysiology of DCI have been investigated [1,8,9]. Although previous studies have demonstrated that EBI is associated with DCI and poor clinical outcomes, the details remain obscure.

We hypothesized that GCE may be linked to post-SAH infection, and that this linkage causes DCI. This study aimed to clarify the association between cerebral edema and post-SAH infection and investigated the effects of this linkage on DCI.

## 2. Materials and Methods

### 2.1. Research Ethics

This cross-sectional study was approved by the Institutional Review Board of our university hospital. We analyzed a prospective database of patients treated with CEA/carotid artery stenting (CAS) at our institution. Informed consent was obtained from all the patients or their guardians using the opt-out method. Formal informed consent was not required, in accordance with the ethical standards of the institutional research committees. Instead, the outline of the study was made available to the public on our homepage, and an option for patients to decline inclusion in the research was provided.

### 2.2. Study Design and Population

**Figure 1:**
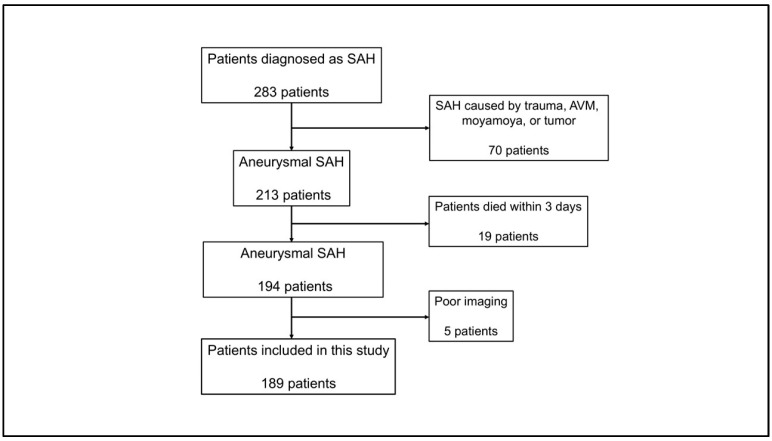
Flow chart of patient selection. Among 283 patients diagnosed with SAH, 94 were excluded for the indicated reasons. Finally, this study included 189 patients. SAH, subarachnoid hemorrhage.

This study was approved by the Institutional Review Board of our institution. Informed consent was acquired from all patients. This observational cohort study included 189 adult patients (≥18 years) diagnosed with aneurysmal SAH at our institution between April 2013 and March 2024. Patients eligible for inclusion in the study underwent non-contrast computed tomography (CT) imaging before aneurysm clipping or endovascular treatment. The exclusion criteria were as follows: (1) SAH related to trauma, brain tumor, moyamoya disease, or cerebral arteriovenous malformation; (2) severe liver dysfunction, leukemia, lymphoma, immune disorder, other hematological diseases, or malignant tumors; (3) death within 3 d of SAH onset; and (4) lack of sufficient radiological or clinical data. The patient inclusion is presented in Figure 1.

### 2.3. Demographic Data and Laboratory Data

Demographic data and data on hypertension, hypercholesterolemia, diabetes mellitus, World Federation of Neurosurgical Societies Scale (WFNS) score at admission, modified Fisher grade [10], aneurysm location, treatment modality, GCE assessed with CT scan on admission, presence of DCI, day of DCI onset, infectious complications, day of infection onset, duration of hospital stay, and clinical outcome at 3 months as assessed using the modified Rankin Scale (mRS) were collected from our registry. Serological data were acquired by collecting blood from the peripheral vein of each patient at admission, 1 day, 9~11 days, and 19~21 days from admission. Serum C-reactive protein (CRP) and neutrophil to lymphocyte ratio (NLR) levels were measured from the obtained blood samples as markers of an inflammatory response [11,12]. The NLR was defined as the absolute neutrophil count divided by the absolute lymphocyte count.

### 2.4. Clinical and Radiographic Assessment

**Figure 2:**
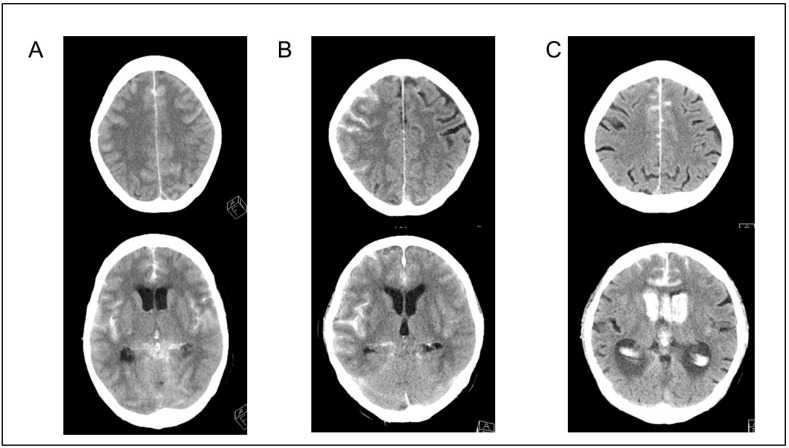
Representative cases. (**A**,**B**) Cases with global cerebral edema (SEBES scores of 4 and 3, respectively). (**C**) A case without global cerebral edema (SEBES score of 0). SEBES, Subarachnoid Hemorrhage Early Brain Edema Score.

All patients underwent non-contrast CT and CT angiography (CTA) imaging or digital subtraction angiography to exclude arteriovenous malformations and moyamoya disease. The Subarachnoid Hemorrhage Early Brain Edema Score (SEBES) was used to evaluate the degree of cerebral edema from CT images obtained at admission as described by Ahn et al. [13]. Briefly, the SEBES was evaluated in two slices at the level of the insular cortex, showing the thalamus and basal ganglion above the basal cistern, and at the level of the centrum semiovale above the level of the lateral ventricle. One point was assigned for the absence of visible sulci caused by effacement or the absence of visible sulci, with disruption of the gray–white matter junction. Each section was scored as 0 for no effacement or a 1 for sulci effacement. Two slices on two sides resulted in a maximum score of 4. In this study, GCE was defined as SEBES 3 or 4. Representative cases are presented in Figure 2.

DCI was defined as a secondary neurological complication that typically occurred 4 d after SAH onset, as described by Vergouwen et al. [14]. Infectious complications were defined as any type of infection diagnosed during the hospitalization period and characterized by a fever in patients with positive microbiological cultures. An axillary temperature of 37.5 °C indicated fever. All outcome variables at 3 months after SAH onset were dichotomized according to clinical outcome as good (mRS 0–3) or poor (mRS 4–6).

### 2.5. Statistical Analysis

Normally distributed data are presented as means ± standard deviation, while categorical variables are presented as frequencies and percentages. Statistical significance was set at *p* < 0.05. Continuous data were analyzed using chi-square tests or Fisher’s exact test. The cumulative incidence of infectious complications was estimated using Kaplan–Meier survival analysis. To compare the risk of infectious complications, the patients were further subdivided for logistic analysis into those with pneumonia, urinary tract infection (UTI), or any type of infection. We then performed logistic regression analysis to compare the risk factors for infection or DCI. Logistic regression is reported as an adjusted common odds ratio (OR) with a 95% confidence interval (CI).

## 3. Results

### 3.1. Demographic and Clinical Characteristics

The baseline characteristics of the included patients are shown in Table 1.

The cohort comprised 189 patients with a mean age of 68.8 ± 11.9 years and was predominantly female (134 patients, 70.9%). At admission, 79 (41.8%) patients presented with WFNS grades 4–5, and 134 (70.9%) patients had a modified Fisher 4. Aneurysms were treated endovascularly in 118 patients (62.4%) and clipped in 71 patients (37.6%). Hydrocephalus was observed on the initial CT in 90 patients (47.6%). Mechanical ventilation was required for 83 patients (43.3%). GCE (SEBES 3 or 4) was present in 88 patients (46.6%), while 101 patients (53.4%) did not have GCE (SEBES < 3). DCI was observed in 58 (30.7%) patients. Of 89 patients who underwent CEA, 31 (34.8%) had hyperuricemia. The demographic data of patients with and without hyperuricemia are presented in Table 1.

### 3.2. Infectious Complications

**Figure 3:**
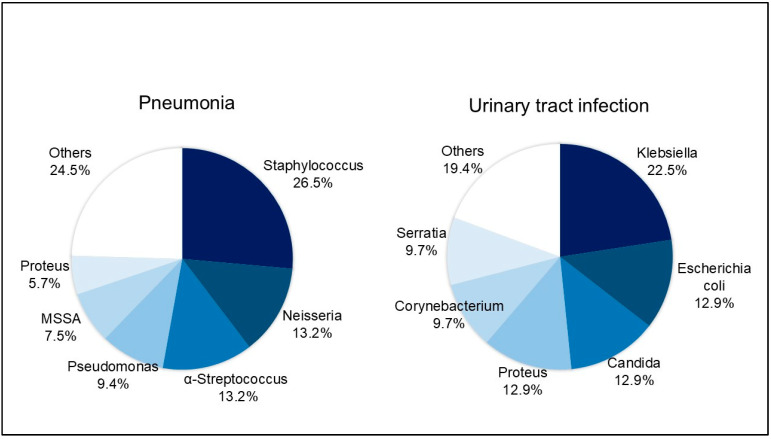
Cultured organisms from patients with pneumonia and urinary tract infection. The most commonly identified organisms were Staphylococcus (26.4%) and Neisseria (13.2%) in sputum culture and Klebsiella (22.6%) and Escherichia coli (13.0%) in urine culture.

Infectious complications occurred in 80 patients (42.3%), with 98 infectious complications developing during the inpatient postsurgical recovery period. Fourteen (7.4%) of these patients showed infections of multiple organ systems. The most frequent infection was pneumonia (n = 53, 54.1%), followed by UTI (n = 31, 31.6%), sepsis (n = 8, 8.2%), meningitis (n = 4, 4.1), and biliary infection (n = 2, 2.0%). Similar to previous studies [15,16,17], 70.0% of the infectious complications were first found to be culture-positive within 10 d of SAH onset. Among the organisms cultured from the patient samples, Staphylococcus (26.4%) and Neisseria (13.2%) were the most prevalent in sputum cultures, while Klebsiella (22.6%) and Escherichia coli (13.0%) were the most commonly detected cultured microorganisms in urine cultures (Figure 3).

**Figure 4:**
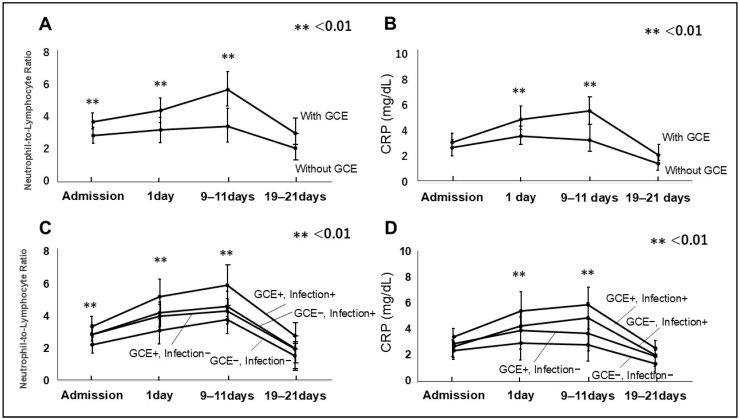
Neutrophil to lymphocyte ratio (NLR) (**A**) and C-reactive protein (CRP) (**B**) in patients with GCE and without GCE are presented. NLR and CRP with GCE on 1 day and 9–11 days after admission were higher than without GCE. NLR and CRP in patients with GCE and infection on 1 day and 9–11 days after admission were higher than in others (**C**,**D**).

NRLs at admission were significant difference among patients with and without GCE (*p* < 0.01, with GCE:3.76 ± 1.12 and without GCE:2.89 ± 0.97, respectively). NRLs on 1 day and 9–11 days in those with GCE were significantly lower than in those without GCE (one day: *p* < 0.01, with GCE: 4.46 ± 1.44, without GCE: 3.25 ± 1.33, 9–11 days: *p* < 0.01, with GCE: 5.76 ± 1.98, without GCE:3.48 ± 1.33, respectively). There were no difference on 19–21 days: *p* = 0.11, with GCE: 3.02 ± 1.41, without GCE; 2.11 ± 1.21, respectively Figure 4A). Similarly, CRP at admission was not different with and without GCE (*p* = 0.23, with GCE 3.12 ± 1.12, without GCE: 2.76 ± 1.31, respectively). CRP on 1 day and 9–11 days with GCE was significantly higher than without GCE (one day: *p* < 0.01, with GCE:4.98 ± 1.66, without GCE: 3.66 ± 1.30, respectively). There were no difference on 9–11 days: *p* < 0.01, with GCE 5.62 ± 1.81, without GCE: 3.36 ± 1.65, respectively Figure 4B). Further, NLRs in patients with GCE and infection at admission, on 1 day, 9–11 days, and 19–21 days were higher than in others (Figure 4C). Similarly, CRP in patients with GCE and infection on 1 day, 9–11 days, and 19–21 days was higher than in others (Figure 4D).

### 3.3. GCE and Infection

**Figure 5:**
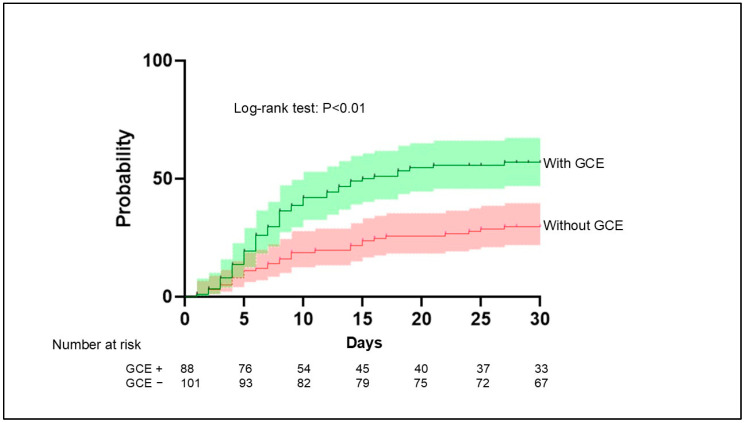
Aggregated survival curves for the probability of infection in patients with and without GCE. Infection occurred significantly more frequently in patients with GCE compared with patients without GCE (*p* < 0.01, log-rank test). GCE, global cerebral edema.

The frequency of infectious complications varied widely between patients with and without GCE, occurring in 50 patients (56.8%) and 30 patients (29.7%), respectively. The results of the Kaplan–Meier analysis demonstrated that infectious complications occurred more frequently in patients with GCE than in those without GCE (*p* < 0.01) (Figure 5).

Logistic regression analysis identified GCE (*p* < 0.001, OR 3.3, 95%CI [1.3–8.6]), higher age (*p* = 0.02, OR 2.5, 95%CI [1.2–4.9]), higher WFNS grade (*p* = 0.01, OR 3.9, 95%CI [1.5–9.5]), and mechanical ventilation use (*p* = 0.04, OR 1.4, 95%CI [1.1–3.9]) as risk factors for infection (Table 2).

### 3.4. DCI Occurrence in Patients with Both GCE and Infection

Comparison of DCI’s occurrence showed that 36 (72.0%) of 50 patients with both GCE and infection had DCI, while 22 (15.8%) of 139 patients with GCE and other conditions had DCI.

**Figure 6:**
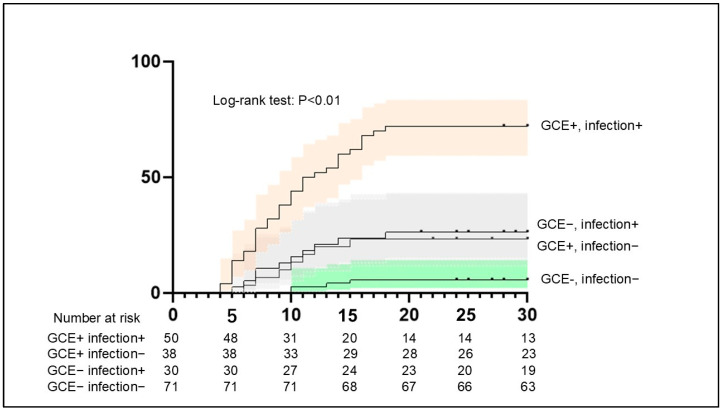
Aggregated survival curves for the probability of DCI between patients with GCE and infection and others. The probability of DCI was significantly higher among patients with GCE and infection compared with others (*p* < 0.01, log-rank test). DCI, delayed cerebral ischemia.

The results of the Kaplan–Meier analysis suggested a higher frequency of DCI among patients with GCE and infection compared with those without GCE (*p* < 0.01) (Figure 6). In 32 of the 36 patients (88.9%) with GCE, infection, and DCI, the infection was confirmed before DCI.

Logistic regression analysis identified age (*p* = 0.03, OR 2.3, 95%CI [1.1–4.6]), WFNS grade (*p* < 0.001, OR 4.5, 95%CI [1.5–9.2]), and GCE + infection (*p* < 0.001, OR 4.1, 95%CI [1.3–8.9]) as independent risk factors for DCI (Table 3).

### 3.5. Clinical Outcomes and Hospital Stay in Patients with GCE and Infection

Clinical outcome was evaluated by mRS at 3 months after SAH onset. Among patients with GCE and infection, 22 (44.0%) and 28 (56.0%) showed good (mRS < 4) and poor (mRS ≥ 4) clinical outcomes, respectively. In contrast, 97 patients (69.8%) had a good clinical outcome and 42 patients (30.2%) had a poor clinical outcome. Poor outcomes occurred significantly more often in patients with GCE and infection compared with other groups (*p* = 0.002). The mean hospital stay in patients with GCE and infection was significantly longer than that in the other patient groups (31.8 ± 18.8 d vs. 24.2 ± 15.0 d, *p* < 0.01).

## 4. Discussion

The principal findings of this study are as follows. First, infectious complications following GCE were associated with DCI, longer hospital stay, and poor clinical outcomes. Therefore, the EBI-DCI chain plays an important role in the postsurgical management of patients with aneurysmal SAH. Second, infectious complications were a common cause of morbidity (42.3%) in patients with SAH. In particular, GCE, older age, poor WFNS grade (4 or 5), and mechanical ventilation were independent risk factors for infection.

### 4.1. Relationship of EBI with Developing Infection

GCE is commonly used as a neuroradiological marker of EBI following SAH. GCE and blood–brain barrier injury that occur in the acute stage (typically within 3 d from ictus) are correlated with DCI and unfavorable outcomes in SAH [18,19]. Our findings demonstrated that GCE plays an important role in the development of infection following SAH. Little is known about the relationship between infection and EBI as represented by GCE. GCE is thought to represent the downstream consequences of EBI, as the cascade of SAH-induced injury mechanisms results in cellular injury, neuroinflammation, blood–brain barrier permeability, and the accumulation of water [20]. Rapid activation of the sympathetic nervous system/hypothalamus–pituitary–adrenal (HPA) axis following an aneurysm rupture may be the first mode of communication between the central nervous system (CNS) and peripheral immune system [21,22]. To date, no direct treatments are available for HPA axis dysfunction. Savarraj et al. suggested that EBI after SAH is associated with increased levels of specific cytokines, such as interleukin (IL)-10, IL-6, and macrophage inflammatory protein (MIP)1β [23]. These cytokines are known biomarkers of immunosuppression, inflammation, and infection [24,25,26]. Furthermore, a cohort study demonstrated continuous suppression of the cellular immune response, including lower CD4+T, CD8+T, and NK cell counts, in patients with high disease severity [27]. The authors also reported that immunosuppression was associated with a high incidence of pneumonia. Zhong et al. suggested a relationship between the inflammatory response and EBI and an association between the inflammatory response and subsequent pneumonia after aneurysmal SAH [28]. These findings support those of the present study showing a link between GCE and infections. Claassen and Park reported that novel treatments for EBI are being studied, which may have larger impacts than interventions aimed at vasospasm or DCI [3]. However, more data are needed to develop strategies to reduce the EBI–infection linkage after aneurysmal SAH.

### 4.2. Infection Following GCE Is Associated with DCI

Various reports have suggested that EBI is related to DCI and poor clinical outcomes; however, the details remain obscure [29,30]. Our data suggest that the GCE–infection–DCI linkage was associated with the poor clinical outcomes of patients with aneurysmal SAH. Perioperative infections are common complications in patients with aneurysmal SAH and are closely associated with longer admissions and higher rates of morbidity and mortality [15,31,32]. SAH has profound effects on systemic immunity related to local immune activation. Systemic immunosuppression occurs soon after early immune activation and is closely associated with infections following SAH. Approximately 30–50% of patients with stroke develop infections; these extracerebral complications are among the leading causes of life-threatening conditions [15,31,33,34]. Therefore, immunosuppression is a potential therapeutic target. However, the extent of systemic inflammatory mediators exacerbating the proinflammatory cascade in brain tissue remains unknown. Our data suggest an infection intermediate between EBI and DCI. Furthermore, the co-existence of EBI, infection, and DCI results in poor patient outcomes. Although experimental studies have proposed many therapeutic agents and methods to improve immunosuppression and infection, these findings have not been reproduced in clinical settings. Thus, novel therapeutic targets against immunosuppression must be further explored, which could help improve outcomes in patients with SAH. The inhibition of EBI-DCI cascades may be an effective means of treating patients with SAH. Previous studies suggested the importance of prediction for DCI of serum and CSF biomarkers linked to oxidative tissue damage, inflammation, and energy breakdown [2,35,36,37,38]. Of them, Messina and colleagues suggested elevated CSF lactate and glucose levels in the first 3 days following aSAH were independent predictors of subsequent DCI-related neurological impairment [36]. They claimed CSF lactate and glucose monitoring may represent a point-of-care test, which could potentially improve the prediction of subacute neurological worsening [36].

### 4.3. Limitations

This study has several limitations. First, it was a single-center study; thus, the findings may not be generalizable to other types of patients. A multi-institutional prospective patient cohort is required to validate our findings across various settings. Second, the sample size was not large enough to include a significant number of patients with certain infection types, such as sepsis and meningitis. A larger multi-institutional cohort study may sufficiently capture such low-frequency events. Third, we did not serially measure cytokine levels, which may provide insights into the peripheral immune response leading to cerebral inflammation. Fourth, we defined the infection based on fever and culture positivoty. However, it is known that infections are occurred subclinically. Further, we evaluated NLR and CRP as markers of inflammation, but we did not explore cytokines or other markers of immune dysregulation, limiting the biological plausibility of the proposed EBI–infection–DCI pathway. In future, we need a prospective study including cytokine and other various markers.

Finally, because of the retrospective analysis of prospectively collected data, only associations, not causalities, can be concluded.

## 5. Conclusions

In conclusion, infectious complications following GCE were strong predictors of DCI, a longer hospital stay, and poor clinical outcomes. Thus, the EBI-DCI chain plays an important role in the postsurgical management of patients with aneurysmal SAH, and the pathophysiology of the EBI–infection–DCI chain warrants close attention.

## Figures and Tables

**Table 1:** Baseline characteristics of included patients.

	SAH Patients
**Number**	**189**
**Demographic**	
Age	68.8 ± 11.9
Sex (female)	134 (70.9%)
Hypertension	59 (31.2%)
Diabetic mellitus	35 (18.5%)
Dyslipidemia	40 (21.2%)
**Patients condition**	
WFNS grade	
1	64 (33.9%)
2	38 (20.1%)
3	8 (4.2%)
4	40 (21.2%)
5	39 (20.6%)
**Radiological finding**	
GCE	88 (46.6%)
Aneurism location	
Acom	59 (31.2%)
ICA	49 (25.9%)
MCA	42 (22.2%)
Distal ACA	12 (6.3%)
Posterior circulation	27 (14.4%)
IVH	33 (17.5%)
ICH	39 (20.6%)
Hydrocephalus	90 (47.6%)
modified Fisher grade	
Grade0	9 (4.8%)
Grade1	11 (5.8%)
Grade2	6 (3.2%)
Grade3	29 (15.3%)
Grade4	134 (70.9%)
**Treatment**	
Endovascular	118 (62.4%)
Clipping	71 (37.6%)
mechanical ventilation	82 (43.3%)
Central venous catheter use	160 (84.7%)
**Morbidity**	
Infection	80 patients with 98 infection
pneumonia	53
UTI	31
sepsis	8
Meningitis	4
Biliary	2
Delayed cerebral ischemic	58 (30.7%)
**Outcime**	
mRS	
Good outcome (mRS 0–3)	119 (63.0%)
Poor outcome (mRS 4–6)	70 (37.0%)

**Table 2:** Multivariate analysis to explore independent risk of infection.

	Infection Positive	Infection Negative	Univariate	Multivariate	
Number (N = 189)	80	109	*p* Value	*p* Value	Odds (95%CI)
**Demographic**					
Age	71.8 ± 10.9	66.9 ± 12.8	0.02	0.03	2.5 (1.2–4.9)
Sex (female)	54	80	0.42		
Hypertension	20	29	0.87		
Diabetic mellitus	15	20	0.98		
Dyslipidemia	19	21	0.48		
**Patients condition**					
WFNS grade (4, 5)	45	34	0.005	0.01	3.9 (1.5–9.5)
**Radiological finding**					
Anrurysm type					
Saccular	75	101	0.98		
Fusiform	5	8			
GCE	50	38	<0.001	0.01	3.3 (1.3–8.6)
Aneurism location					
Acom	28	31	0.86		
ICA	20	29			
MCA	18	24			
Distal ACA	4	8			
Posterior circulation	10	17			
IVH	15	18	0.7		
ICH	22	17	0.07	0.09	
Hydrocephalus	40	50	0.66		
modified Fisher grade					
Grade0	4	5	0.98		
Grade1	5	6			
Grade2	3	3			
Grade3	11	18			
Grade4	57	77			
**Treatment**					
Aneurysm treatment					
Endovascular	55	63	0.13	0.19	
Clipping	25	46			
Mechanical ventilation use	45	37	0.003	0.04	1.4 (1.1–3.9)
Central venous catheter use	70	90	0.42		

**Table 3:** Multivariate analysis to explore independent risk of DCI.

	DCI Positive	DCI Negative	Univariate	Multivariate	
NUMBER (N = 189)	58	131	*p* Value	*p* Value	Odds (95%CI)
**Demographic**					
Age	70.8 ± 10.9	67.9 ± 12.8	0.03	0.03	2.3 (1.1–4.6)
Sex (female)	40	94	0.73		
Hypertension	15	34	0.98		
Diabeticmellitus	11	24	0.98		
Dyslipidemia	15	25	0.34		
**Patients condition**					
WFNS grade (4, 5)	40	39	<0.001	<0.001	4.5 (1.5–9.2)
**Radiological finding**					
Anrurysm type					
Saccular	54	122	0.98		
Fusiform	4	9			
GCE + infection	36	22	<0.001	<0.001	4.1 (1.3–8.9)
Aneurism location					
Acom	20	39	0.74		
ICA	14	33			
MCA	15	27			
Distal ACA	2	10			
Posterior circulation	7	20			
IVH	13	20	0.3	0.18	
ICH	14	22	0.24	0.11	
Hydrocephalus	28	62	0.95		
modified Fisher grade					
Grade0	3	6	0.98		
Grade1	4	7			
Grade2	2	4			
Grade3	8	21			
Grade4	45	89			
**Treatment**					
Aneurysm treatment					
Endovascular	40	78	0.33		
Clipping	19	52			
Mechanical ventilation use	30	52	0.4		
Central venous catheter use	50	110	0.83		

## Data Availability

The data analyzed in the current study are available from the corresponding author upon reasonable request.
